# Type of hormonal treatment administered to induce vitellogenesis in European eel influences biochemical composition of eggs and yolk-sac larvae

**DOI:** 10.1007/s10695-021-01042-4

**Published:** 2022-01-19

**Authors:** E. Benini, S.N. Politis, A. Nielsen, S. R. Sørensen, J. Tomkiewicz, S. Engrola

**Affiliations:** 1 Technical University of Denmark, National Institute of Aquatic Resources, Kgs. Lyngby, Denmark; 2 Centre of Marine Sciences, Universidade Do Algarve, Faro, Portugal

**Keywords:** Assisted reproduction, Pituitary extract, Egg and larval quality, FAA, EFA, Lipids, Protein

## Abstract

Egg biochemical composition is among the main factors affecting offspring quality and survival during the yolk-sac stage, when larvae depend exclusively on yolk nutrients. These nutrients are primarily embedded in the developing oocytes during vitellogenesis. In aquaculture, assisted reproduction procedures may be applied enabling gamete production. For the European eel (*Anguilla anguilla*), reproductive treatment involves administration of pituitary extracts from carp (CPE) or salmon (SPE) to induce and sustain vitellogenesis. In the present study, we compared the influence of CPE and SPE treatments on offspring quality and composition as well as nutrient utilization during the yolk-sac stage. Thus, dry weight, proximal composition (total lipid, total protein), free amino acids, and fatty acids were assessed in eggs and larvae throughout the yolk-sac stage, where body and oil-droplet area were measured to estimate growth rate, oil-droplet utilization, and oil-droplet utilization efficiency. The results showed that CPE females spawned eggs with higher lipid and free amino acid contents. However, SPE females produced more buoyant eggs with higher fertilization rate as well as larger larvae with more energy reserves (estimated as oil-droplet area). Overall, general patterns of nutrient utilization were detected, such as the amount of total lipid and monounsaturated fatty acids decreasing from the egg stage and throughout the yolk-sac larval stage. On the contrary, essential fatty acids and free amino acids were retained. Notably, towards the end of the yolk-sac stage, the proximal composition and biometry of surviving larvae, from both treatments, were similar.

## Introduction

The production of good-quality eggs relies on the optimal progression of oogenesis, where the coordinated assembly of developing eggs is controlled by an interplay of endocrine and intra-ovarian factors, a process that can take a year or more (Tyler and Sumpter 1996). In vertebrates, pituitary gonadotropins regulate oogenesis through stimulation of sex steroid synthesis by follicle cells surrounding the developing oocytes (Nagahama and Yamashita 2008). The pituitary produces two types of gonadotropin, follicle-stimulating hormone (FSH) and luteinizing hormone (LH) (Brooks et al. 1997)*.* In oviparous fishes, FSH acts on follicular granulosa and thecal cells, stimulating the synthesis of estradiol-17β (Suzuki et al. 1998), which among other stimulates the production of a glycophosphate lipoprotein (vitellogenin) by the liver (Lubzens et al. 2010; Li and Zhang 2017; Reading et al. 2018). Vitellogenin is transported to the ovary and incorporated into the developing oocytes as cytoplasmic yolk granules or globules (Ohkubo et al. 2008), while it is considered the major source of amino acids, lipids, and calcium necessary for embryonic development (Brooks et al. 1997). On the other side, the role of LH primarily relates to the final maturation of the follicle, stimulating the production of maturation-inducing hormone (MIH) and maturation-promoting factor (MPF), leading to ovulation (Nagahama and Yamashita 2008).

The composition of eggs is among the most recognized factors affecting offspring quality and survival, where the nutrient-bearing yolk portion has the primary function to nourish the developing embryos and larvae (Brooks et al. 1997; Bobe 2015). These nutrients, along with other cytological constituents, originate from maternal resources being deposited in the oocytes during the secondary growth phase (Reading et al. 2018). Overall, these patterns are similar in oviparous fishes; however, the exact composition of fish eggs is species-specific and the precise sequence of consumption and utilization by the developing embryo and larva varies.

Fish eggs can be classified into two energetic categories, i.e. without or with oil droplets, referred to Type I and Type II eggs (Rønnestad et al. 2013). Offspring of fish species with Type II eggs, including the European eel (*Anguilla anguilla*), acquire about half of their energy from amino acids and about half from lipids, in particular monounsaturated fatty acids (MUFAs). While MUFAs are utilized for energy generation (Rainuzzo et al. 1994; Finn et al. 1994; Sargent et al. 1999), yolk saturated fatty acids (SFAs) and polyunsaturated fatty acids (PUFAs) are retained to sustain development of the organism until the feeding stage (Kamler 2008). Thus, adequate amounts of PUFAs need to be present in the eggs ( Bell and Sargent 2003), as suboptimal levels of essential fatty acids (EFAs), such as arachidonic acid (ARA), eicosapentaenoic acid (EPA), and docosahexaenoic acid (DHA), can cause poor growth, skeleton deformities, and immune deficiency leading to increased mortality (Izquierdo 2005). Overall, the amount and composition of EFAs in the fish eggs and yolk-sac larvae is strongly influencing offspring quality (fertilization and hatching rate) and early larval survival (Tocher 2010).

Proteins are also fundamental constituents of fish eggs among other providing amino acids necessary for organ and muscle growth as well as dispensing energy via catabolic processes (Rønnestad et al. 1993). Thus, the yolk-sac comprises a pool of free amino acids (FAAs), representing around 50% of the amino acids present in marine fish eggs (Fyhn 1990). Already during final maturation of the oocyte, FAAs serve as osmotic effectors responsible for the influx of water and hydration that is required for neutral buoyancy of the eggs (Seoka et al. 2004). Later during yolk resorption, the FAA pool is depleted and reaches low levels at first feeding (Rønnestad et al. 1999). Thus, during the pre-feeding period, larvae are highly dependent on the FAA pool as fuel in the energy dissipation, as shown for fish species such as lemon sole, *Microstomus kitt*, Atlantic halibut, *Hippoglossus hippoglossus* and cod, *Gadus morhua* (Rønnestad et al. 1993; Conceição and Tandler 2018).

In aquaculture, the control of gamete quality and successful offspring development is of immense importance (Migaud et al. 2013). Here, maternal dietary requirements must be met to obtain high-quality eggs, including adequate amounts of EFAs, in particular for marine fish that are incapable of synthesizing and prolonging PUFAs (Bell and Sargent 2003). Moreover, assisted reproduction procedures, including exogenous hormonal therapies, are often necessary to produce offspring in captivity (Bobe and Labbé 2010; Mylonas and Zohar 2010), which may influence oogenesis and egg quality. In the case of European eel, where closing the life cycle in captivity is targeted to sustain aquaculture, assisted reproduction protocols need to be applied to overcome a natural inhibition of sexual maturation in both sexes (Vidal et al*.* 2004; Tomkiewicz 2012; Mordenti et al. 2019). Female reproduction protocols involve repeated administration of pituitary extracts of carp (CPE) or salmon (SPE) to induce and sustain vitellogenesis, while administration of a maturation-inducing steroid is needed to complete oocyte maturation and ovulation (Palstra et al. 2005; Da Silva et al. 2018; Kottmann et al. 2021). Recently, Kottmann et al. (2020a), studying the differential impact of CPE and SPE administration on egg quality and embryonic developmental competence, reported higher proportions of buoyant eggs and embryonic survival in offspring from SPE-treated females compared to CPE females. Thus, different content and composition of hormones in the pituitary glands and/or a species-specific affinity of eel receptors to carp and/or salmon hormones may influence egg quality (Schmitz et al. 2005).

In this context, we hypothesized that different hormonal therapies may influence oocyte development, resulting in variable egg biochemical composition and leading to differences in nutrient composition of eggs and yolk-sac larvae. As such, the objective of this study was to compare the effect of two maternal hormonal treatments (i.e. SPE vs. CPE) on the biochemical composition of European eel offspring and larval utilization of resources throughout early development. Fertilized eggs were sampled 4 h post-fertilization (hpf), while larvae were sampled at regular intervals from hatch to the first-feeding stage (at 0, 4, 8, and 13 days post-hatch (dph)). Biochemical variables measured included dry weight, total lipid, fatty acid, total protein, and free amino acid composition, while morphometric larval quality indicators included body area, oil-droplet area, growth rate, and oil-droplet absorption/utilization rate.

## Material and Methods

### Broodstock and gamete production

Wild-caught European eel females from a brackish lake (Saltbæk Vig, Denmark) were transported to the EEL-HATCH research facility of the Technical University of Denmark, located in Hirtshals, Denmark. Upon arrival, fish were randomly distributed at a density of ~ 15 fish/tank into replicated ~ 1150-L polyethylene tanks equipped with a recirculating aquaculture system (RAS), under a continuous flow rate of ~ 10–15 L min^−1^, low-intensity light (~ 20 lx), and 12-h day:12-h night photoperiod (Tomkiewicz 2012). Male eels were obtained from a commercial eel farm (Royal Danish Fish) located in Hanstholm (Denmark) and reared in a similar, but smaller RAS (~ 450L capacity) at a density of 15–22 fish/tank under similar ambient conditions. During the experiment, water temperature and salinity were maintained at ~ 20 °C and ~ 36 psu, respectively, while feeding was ceased as eels naturally undergo a fasting period from the onset of the pre-pubertal silvering stage. Females were randomly divided into two groups. After two weeks of acclimatization, one group received weekly injections of salmon pituitary extract (SPE, 18.75 mg kg^−1^ initial body weight, Argent Chemical Laboratories, Washington, USA), while the second group received weekly injections of carp pituitary extract (CPE, same dose, and same supplier) to induce vitellogenesis (Kottmann et al. 2020a). For completion of final maturation and ovulation, an additional injection of PE was given in combination with administration of 17α,20ß-dihydroxy-4-pregnen-3-one (DHP, Sigma-Aldrich, St. Louis, MO, USA) at 2.0 mg kg^−1^ present body weight, with stripping of eggs 12–14 h after injection (Palstra et al. 2005; Kottmann et al. 2021). The male eels received weekly injections of human chorionic gonadotropin (hCG, Sigma-Aldrich, Missouri, USA) at 150 IU/fish (Pérez et al. 2000). An additional injection of hGC was given to male eels ~ 12 h prior to spawning (Koumpiadis et al. 2021). Stripped milt from four to five males per female (Benini et al. 2018) was pipetted into an immobilizing medium (P1 medium, Peñaranda et al. 2010) at a standardized concentration of 1:99 and used for fertilization within 4 h of collection (Butts et al. 2014; Sørensen et al. 2013).

### Fertilization and embryonic incubation

Stripped eggs from each female were fertilized by a pool of standardized milt. Upon mixing of gametes, artificial sea water (ASW), i.e. tap water filtered through a reverse osmosis filtration system (Vertex Puratek 100 gpd RO/DI, Vertex Technologies Inc., CA, USA) salted up to 36 psu with Reef Salt (Red Sea, Red Sea International, Eilat, Israel), was added for activation followed by incubation at temperature of 20 °C (Sørensen et al. 2016a). Initially, the eggs were incubated in 15 L of ASW for 1 h, from where the buoyant egg layer was gently moved into new 15 L of ASW. Two hours post-fertilization (hpf), buoyant eggs were transferred to 60-L conical egg incubators and supplied with conditioned and filtered seawater at a flow through rate of ~ 350 mL min^−1^ (Politis et al. 2018b). Gentle aeration was added after ~ 10 hpf, while temperature was lowered to 18 °C for optimized embryonic development (Politis et al. 2017). Light was kept at a low intensity of ~ 10 lx (Politis et al. 2014), and sinking dead eggs were purged from the bottom valve of each incubator. At ~ 52 hpf aeration was stopped and hatching occurred at ~ 56 hpf.

### Larval culture

After hatch, each batch of larvae was transferred into separate 80-L tanks connected to a RAS system consisting of biofilter, trickle filter, UV (ProCristal UV-C 11 W, JBL GmbH & Co. Neuhofen, Germany), protein skimmer (AquaMedic 5000 single 6.0, Bissendorf, Germany), and reservoir for top-up. Temperature and salinity were maintained at 18 °C and 36 psu, respectively, with low-intensity illumination (Politis et al. 2014). On 4 dph, a fraction of each batch of larvae was transferred to three 8-L Kreisel tanks (~ 1000 larvae each tank). Each Kreisel was connected to a RAS, kept at 18 psu (Politis et al. 2018a; 2021) and 18 °C (Politis et al. 2017), while larvae were reared in total darkness and monitored under low-intensity illumination (Politis et al. 2014). Flow rates in tanks were kept at ~ 420 mL min^−1^. The experiment continued until the larvae reached the first-feeding stage (13 dph).

### Egg production, fertilization success, hatching success, and larval biometry

The weight of stripped eggs was recorded prior to fertilization, while ~ 30 min post-fertilization, the amount of buoyant eggs (%) was determined in a 25-mL volumetric column (Tomkiewicz 2012).

At 4 hpf, a sample of eggs (n ~ 100 eggs) was obtained and photographed using a stereomicroscope (SMZ1270i, Nikon Corporation, Japan) with a mounted camera (Digital Sight DS-Fi2, Nikon Corporation, Japan). Subsequently, digital images were analysed applying NIS-Elements-D analysis software (Version 3.2, Nikon Corporation, Japan). Using the 4-cell stage as criterion (Sørensen et al. 2016b), fertilization success was calculated as the percentage of fertilized eggs divided by the total number of eggs imaged. In order to estimate the embryonic survival at 24 and 48 hpf, the number of embryos was calculated in 3 × 15 ml water samples collected from the incubators. For the estimation of hatching success, subsamples of ~ 100 embryos were collected from the incubators at 48 hpf and inserted into 200-mL sterile tissue culture flasks (VWR, Denmark) filled with culture water enriched with rifampicin and ampicillin (each 50 mg L^−1^, Sigma-Aldrich, Missouri, USA) (Sørensen et al. 2014). Approximately 12 h after hatching, the number of larvae and unhatched embryos was recorded to assess hatching success (%).

### Sampling and data collection

**Fig. 1:**
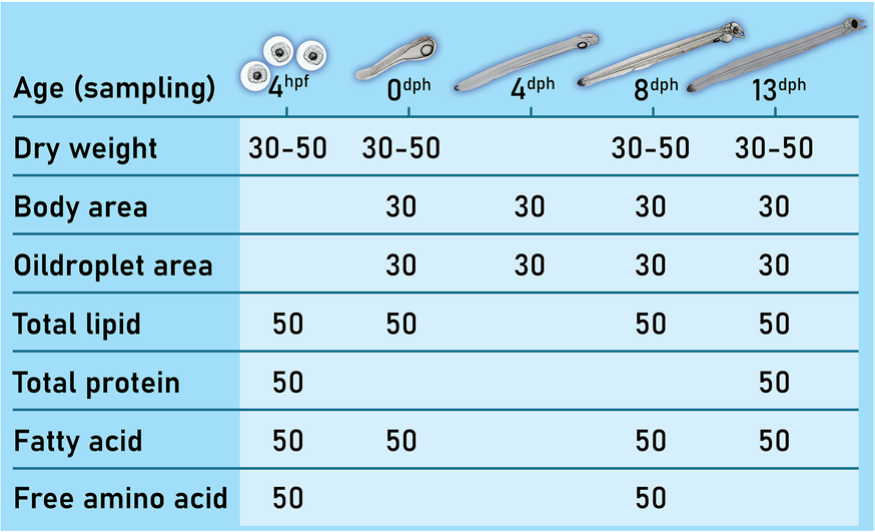
– Overview over measured variables included in the study and total number of specimens sampled and analysed per age and variable

The offspring from each of the 11 parental crosses were sampled at 4 hpf, at hatch and at regular intervals throughout the endogenous feeding larval stage until the first-feeding stage (0, 4, 8, 13 dph). Figure 1 provides a sampling overview and the number of eggs and larvae sampled for the various analyses.

For each batch, 10 larvae from each of 3 replicates were photographed at each sampling point (hatch, 0, 4, 8 and 13 dph) for biometric analyses. Body area (mm^2^) and oil-droplet area (mm^2^) were measured for each larva. Larval growth (GR) and oil-droplet utilization (ODU) were measured from the change in body and oil-droplet area from hatching (0 dph) until first feeding (13 dph) (Politis et al. 2017). Moreover, the oil-droplet utilization efficiency (ODUE) was calculated by dividing the increase in body area between 0 and 13 dph by the corresponding decrease of yolk area.

### Dry weight and biochemical composition of eggs and larvae

Eggs and larvae were collected at 4 hpf and at 0, 8, and 13 dph, euthanized with MS-222, washed in deionized water, snap-frozen in liquid nitrogen and stored at -80 °C until further analysis (Christ Beta 2–15, Martin Christ Gefriertrocknungsanlagen GmbH, Germany). After freeze-drying, samples were weighed using a microbalance (Mettler-Toledo MT5, Mettler-Toledo A/S, Denmark). For the analyses of total protein, FAAs, and lipids, samples were transported to the Centre of Marine Sciences (Faro, Portugal). Quantification of FA concentration was conducted at the Technical University of Denmark (Kgs. Lyngby, Denmark).

For lipid extraction of eggs (0.1 ml) and larvae (50 larvae per sample) a homogeneous mixture of chloroform, methanol, and distilled water (2:2:1.8) was used, following a modified method of Bligh and Dyer (1959). The lipid extracts were heated at 60 °C over night, and the lipid content was determined by gravimetry after evaporation of chloroform. Lipids were transferred and weighed on pre-weighed vials. Finally, the extracts were weighed on a Mettler Toledo MT5 scale (Mettler-Toledo A/S, Glostrup, Denmark; d = 0.1 μg) and the amount of total lipid was calculated as the percentage of dry weight (mg ind^−1^).

The protein content of eggs and larvae was determined from 1 mg of freeze-dried samples and obtained by applying the Kjeldahl method with a conversion factor of 6.25 as described in AOAC (2006). Finally, protein content was calculated as a percentage of dry weight. Due to the small size of the samples, the analysis of protein content on 0 and 8 dph was not reliable and therefore not further considered in this study.

Lipids for estimation of FA composition were extracted from fertilized eggs and larvae at 0, 8, and 13 dph, following Folch et al. (1957). A 1 mL mixture of chloroform/methanol (2:1 v/v) was added to the samples with 40 μL internal standard of methyl tricosanoate (C23:0) in chloroform. Samples were placed in an ice-water bath, sonicated in a 2510 Branson ultrasound cleaner for 25–30 min, and subsequently kept for 24 h at − 20 °C to extract lipids. The sample was then transferred to 1.5-mL auto-sampler vials with Butyl/PFTE septa screw caps, and all liquid evaporated at 60 °C by applying a flow of nitrogen from a needle into the mouth of the vial for ~ 20 min with a nine-port Reacti-Vap Evaporator in a Pierce Reacti-Therm heating module. Thereafter, 1 mL of a toluene, methanol, and acetyl chloride solution (40: 50: 10) was added to the sample and heated for 2 h at 95 °C. The vials then received 0.5 mL of aqueous NaHCO_3_. After shaking the sample, the layer containing the FA methyl esters (FAME) was removed. The extraction was repeated twice, by the addition of 0.5 mL heptane. The combined sample was added to 2-mL screw top vials with silicone/PFTE septa and evaporated at 60 °C with additional nitrogen flow. Esterified samples were analysed by gas chromatography (Thermo Scientific Trace 1300), and analytes were detected by a single-quadrupole mass spectrometer (Thermo Scientific ISQ 7000). Split less injection was used and an inlet temperature of 220 °C. The carrier gas was helium and was set at a constant flow of 1.2 mL min^−1^ throughout the run. Separation of the FAME was achieved using a Thermo Scientific Trace GOLD TG-5MS column (length: 30 m; diameter: 0.25 mm; film thickness: 0.25 µm). The following temperature gradient was used for the separation: the initial temperature of 60 °C was held for 1 min, followed by an increase to 150 °C at 40 °C min^−1^, and then slowly raised to 220 °C at 2.5 °C min^−1^. The temperature was held at 220 °C for 14 min, raised to 300 °C at 40 °C min^−1^, and finally held at 300 °C for 5 min. Ionization was undertaken by electron impact, and the mass range was set to 10–800 m*/z*. The MS transfer line was held at 250 °C, while the ion source temperature was set to 300 °C. FAME identification was undertaken by reference to an external standard consisting of a mixture of FAMEs (Supelco, F.A.M.E. Mix, C4-C24, 18,919-1AMP). Identification of samples was confirmed with the NIST library (similarity index threshold of 800). The FAME composition was determined as a proportion of the total integrated peak areas of all detected peaks. Detector linearity (R^2^ > 0.92) over the observed ranges of FAMEs was confirmed by external calibration curves of each of the measured FAMEs of the standard mixture.

FAA content was determined in fertilized eggs and larvae at 8 dph. After homogenization of freeze-dried samples in 0.1 M HCl on ice, samples were centrifuged at 1,500 g at 4 °C for 15 min and supernatant deproteinized by centrifugal ultrafiltration (10-kDa cut-off, 2500 × g at 4 °C for 20 min). All samples were pre-column derived with Waters AccQ Fluor Reagent (6-aminoquinolyl-N-hydroxysuccinimidyl carbamate) using the AccQ Tag method (Waters, USA). All analyses were performed by ultra-high-performance liquid chromatography (UPLC) on a Waters Reversed-Phase Amino Acid Analysis System, using norvaline as an internal standard. Instrument control, data acquisition, and processing were achieved using Waters Empower software.

### Statistical analyses

Data from the experiment were analysed through a series of ANOVA models (Keppel 1991) using R Studio Software (Version 1.3.959, *RStudio: Integrated Development for R. RStudio, PBC, Boston, MA*). Prior to analysis, residuals were tested for normality (Shapiro–Wilk test) and homogeneity of variances (plot of residuals vs. fitted values). Data deviating from normality or homoscedasticity were log10 transformed. Alpha was set at 0.05. Tukey's analysis was used to compare least-squares means between treatments. Family ID (individual females and their offspring) was considered random in all models. When no significant interaction was detected for the tested dependent variable, the model was re-run with the interaction effect removed, analyzing main effects separately. Hence, we analysed the main effects of hormonal treatment (CPE and SPE) on offspring quality in terms of the various dependent variables (Table 1) in relation to stage of development (4 hpf and 0, 4, 8, or 13 dph). A series of pairwise t tests were used to calculate differences between groups (CPE vs. SPE), when only the effect of treatment was influencing the data.

**Table 1:** - Female data and reproductive success in relation to hormonal treatments (CPE vs SPE). Mean values (± standard deviation) for each group for all the characteristics of European eel broodstock, eggs, and embryos are also shown. * represent significant differences (p<0.05)

Female ID	Treatment	Length (cm)	Weight (g)	Injection (n)	Batch (g)	Buoyant eggs (%)	Fertilization rate (%)	Survival 24 hpf (%)	Survival 48 hpf (%)	Hatching rate (%)
D01D	CPE	74	720	10	1006	50	91.3	72.8	72.5	72.5
CDC6	CPE	65	458	14	9000	99	56.1	NA	42,7	39.1
CAFB	CPE	75	836	14	751	100	45.3	19.9	12.0	9.3
D1DB	CPE	83	1230	15	813	80	38.7	33.5	23.8	15.9
D08B	CPE	72	682	16	616	80	58.2	58.2	27.2	3.0
Mean CPE		73.9 (± 6.3)	785.2 (± 283.9)	13.8 (± 2.3)	817 (± 147.6)	81.8 (± 20)	57.9 (± 20.3)	46.13 (± 23.8)	34.84 (± 23.7)	27.93 (± 28.4)
CBD2	SPE	55.5	320	17	763	90	80.9	53.6	29.1	7.9
DCB9	SPE	72	832	11	817	100	92.7	70.3	47.5	43.9
C96C	SPE	67	626	11	780	90	74.1	67.9	40.9	10.7
CB1A	SPE	72	790	12	780	95	67.9	61.8	35.6	33.4
CF9A	SPE	77	880	12	523	50	49.2	45.5	13.9	7.2
CBAF	SPE	71	784	12	791	90	36.9	32.2	14.6	13.3
Mean SPE		69 (± 7.4)	705.29 (± 207.2)	12.50 (± 2.3)	742 (± 108.9)	85.80* (± 18)	66.95* (± 20.6)	55.27 (± 14.7)	30.24 (± 13.8)	19.39 (± 15.43)

## Results

### Reproductive success and offspring development

Table 1 presents data of the 11 females on their reproductive success in relation to hormonal treatment. Neither the length and initial weight of the females nor the amount of eggs produced per female were significantly different. However, the relative amount of buoyant eggs and relative fertilization success were significantly higher in SPE compared to CPE batches (for both p < 0.01), while embryonic survival and hatching success did not differ between the two groups.

### Biometry

**Fig. 2:**
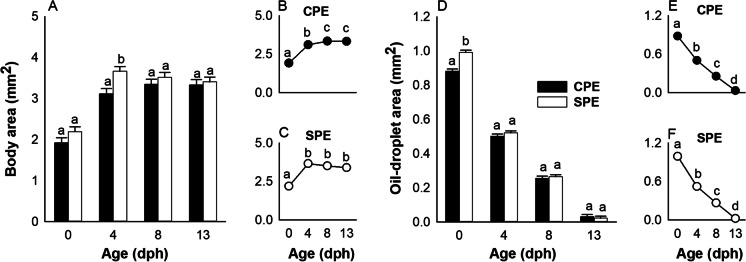
– Association between maternal hormonal treatment (CPE vs SPE), body area (A, B, and C), and oil-droplet area (D, E, and F) of European eel larvae. Values represent means (± SEM) among batches. Different letters indicate significant differences (p < 0.05)

The body area of larvae increased from 2.2 ± 0.3 mm^2^ at hatch (0 dph) to 4.3 ± 0.7 mm^2^ at the end of the endogenous feeding stage (13 dph). The statistical analysis detected a treatment × age interaction (p < 0.0001). Thus, the model was decomposed to a series of reduced ANOVA models to determine the effect of treatment at each age (Fig. 2 A) and the effect of age for each treatment (Fig. 2 B and C). At hatch and 4 dph, the body area of SPE larvae was significantly larger than the body area of larvae from the CPE treatment (p < 0.05). This difference diminished with age, resulting in no significant difference between the larvae sampled from the two treatments at 8 and 13 dph (p > 0.05). Concomitantly, the oil-droplet area (mm^2^) decreased from 0.942 ± 0.07 mm^2^ at 0 dph to 0.0262 ± 0.015 mm^2^ at 13 dph. Also here, the treatment × age interaction was significant (p < 0. 0001), so the model was decomposed to determine the effect of treatment at each age (Fig. 2 D) and the effect of age for each treatment (Fig. 2 E and F). In particular, larvae from the SPE treatment had a significant (p < 0.001) larger oil-droplet area (0.993 ± 0.001 mm^2^) at hatch compared to larvae of the same age from the CPE treatment (0.882 ± 0.001 mm^2^). Later in development (at 4, 8, and 13 dph), this difference between offspring from the two treatment groups was no longer detectable (p > 0.05).

### Growth rate, oil-droplet utilization rate, and oil-droplet utilization efficiency

**Fig. 3:**
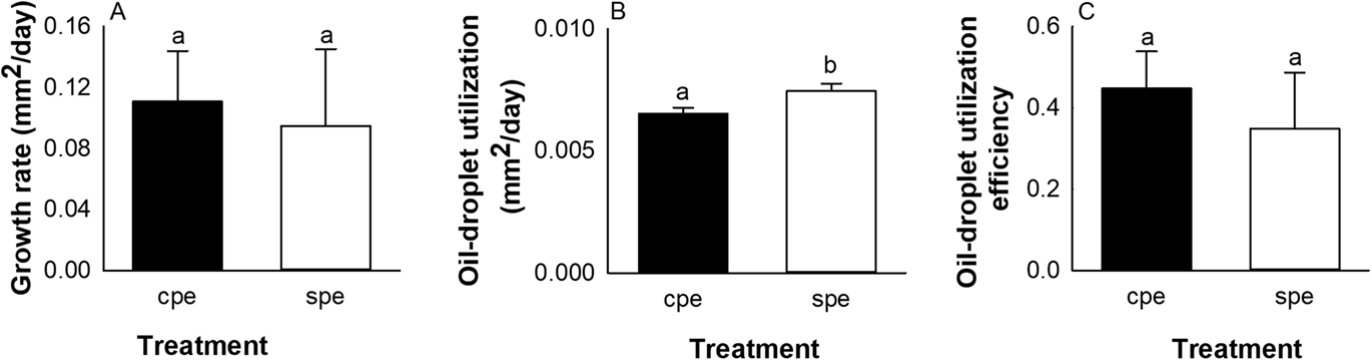
– Effect of maternal hormonal treatment (CPE vs SPE) on growth rate (A), oil-droplet utilization (B), and oil-droplet utilization efficiency (C) in European eel larvae between hatch and the end of the endogenous feeding stage (13 dph). Values represent means (± SEM) among batches. Treatments with different letters are significantly different (p < 0.05)

Growth rate (mm^2^/day), calculated over a period of 13 days, ranged between families from 0.021 ± 0.013 mm^2^/day to 0.16 ± 0.006 mm^2^/day and did not differ between treatment groups (Fig. 3 A). Oil-droplet utilization (mm^2^/day) was significantly influenced by the maternal treatment (p < 0.001), with SPE batches having a significantly higher daily oil-droplet utilization than CPE ones (Fig. 3 B). However, oil-droplet utilization efficiency was not influenced by treatment (Fig. 3 C).

### Dry weight, total lipids, and total protein

**Fig. 4:**
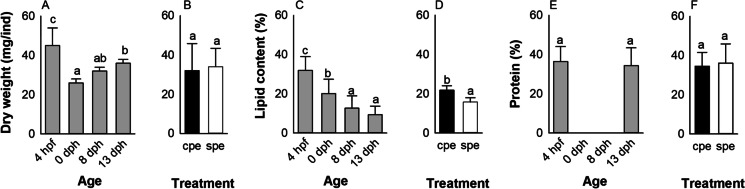
– Effect of maternal hormonal treatment (CPE vs SPE) and age on dry weight (A, B), total lipid (C,D), and total protein (E,F) in eggs and larvae of European eel. Values represent means (± SEM) among batches. Different letters represent significant differences (p < 0.05)

Dry weight showed no treatment × age interaction and was not influenced by treatment (Fig. 4 B), but varied during development (p < 0.001). Here, dry weight significantly (p < 0.0001) reduced by ~ 50% from 0.045 ± 0.009 mg/individual at the egg stage to 0.026 ± 0.003 mg/individual at hatch. After hatch, dry weight of larvae gradually increased reaching 0.032 ± 0.007 mg/individual at 8 dph and 0.036 ± 0.009 mg/individual at 13 dph. No significant difference was observed between 0 and 8 dph nor between 8 and 13 dph (Fig. 4 A).

On the other hand, total lipid content in offspring was significantly affected by maternal treatment (p = 0.0071) and age (p < 0.0001), while no significant treatment × age interaction was found. Total lipid content decreased from 37.62 ± 2.5% in fertilized eggs to 6.94 ± 2.3% in 13-day-old larvae. The total lipid content was significantly (p < 0.01) different between the egg stage and larvae at hatch as well as between hatch and 8 dph, while no significant difference was observed between 8 and 13 dph (Fig. 4 C and D). Moreover, CPE eggs and larvae contained more lipids (21.78 ± 2.121%) than their SPE counterparts (15.84 ± 2.324%).

In addition, total protein content measured in fertilized eggs and first-feeding larvae (13 dph) was not affected by treatment or age and the overall total protein content was stable around 35% throughout the endogenous stage (Fig. 4 E).

### Free amino acids (FAAs) analysis

FAA content was analysed in fertilized eggs (4 hpf) and in larvae at 8 dph (Table 2). The FAA pool was divided into essential (EAA) and non-essential amino acids (NEAAs). The sum of EAAs increased significantly with age (p < 0.001), but was not affected by treatment (p = 0.364). EAAs were significantly affected by the treatment × age interaction (p < 0.01), including histidine, isoleucine, leucine, phenylalanine, threonine, and valine. In order to evaluate the effect of treatment at each stage, a series of one-way ANOVAs were performed. A significant difference in EAAs between treatments was present at the fertilized egg stage (p < 0.001), while this difference was no longer detectable in larvae at 8 dph (p > 0.05). On the other hand, levels of arginine, lysine, and methionine were significantly (p < 0.01) affected by age, but not by treatment. Moreover, tryptophan showed no treatment × age interaction, but was influenced by both, treatment (p = 0.03) and age (p < 0.001). Here, the amount of tryptophan increased with age and SPE eggs and larvae contained more tryptophan than CPE ones.

**Table 2:** – Free amino acid values (± SD), essential in fertilized eggs, and yolk-sac larvae at 8 dph for two hormonal treatments (CPE and SPE) in European eel. Different lower and upper case letters represent significant statistical difference (p < 0.05)

	***Fertilized eggs (4 hpf)***		***Larvae (8 dph)***		***Model on fertilized eggs and larvae***		
	***CPE***	***SPE***	***CPE***	***SPE***	***Age x Treatment*** ***p-value***	***Age*** ***p-value***	***Treatment*** ***p-value***
**Arginine**	16.64 ± 4.76	14.63 ± 3.32	32.79 ± 2.66	34.34 ± 2.40	0.0755	< 0.00001	0.65311
**Histidine**	3.76 ± 1.11 ^a^	1.44 ± 0.24 ^b^	7.00 ± 0.77 ^a^	7.01 ± 0.51 ^a^	0.00118	-	-
**Isoleucine**	1.68 ± 0.15 ^a^	2.95 ± 0.23 ^b^	4.69 ± 0.51 ^a^	5.07 ± 0.38 ^a^	0.0074	-	-
**Leucine**	3.17 ± 0.33 ^a^	4.71 ± 0.46 ^b^	3.8 ± 0.43 ^a^	3.88 ± 0.25 ^a^	0.00026	-	-
**Lysine**	3.95 ± 0.41	4.99 ± 1.47	7.15 ± 0.84	7.52 ± 0.57	0.41078	< 0.0001	0.09394
**Methionine**	1.21 ± 0.14	1.30 ± 0.32	1.82 ± 0.24	1.82 ± 0.19	0.67026	< 0.00001	0.6115
**Phenylalanine**	2.33 ± 0.23 ^a^	1.5 ± 0.35 ^b^	4.65 ± 0.58 ^a^	4.9 ± 0.38 ^a^	0.00575	-	-
**Threonine**	2.69 ± 1.20 ^a^	5.96 ± 2.18 ^b^	12.9 ± 1.42 ^a^	12.6 ± 0.78 ^a^	0.01272	-	-
**Valine**	2.40 ± 0.37 ^a^	0.54 ± 0.05 ^b^	0.77 ± 0.06 ^a^	0.86 ± 0.07 ^a^	< 0.00001	-	-
**Tryptophan**	1.43 ± 0.027 ^a^	1.56 ± 0.15 ^b^	2.61 ± 0.23 ^a^	2.92 ± 0.24 ^b^	0.37075	< 0.00001	0.03068
**ΣEAA**	**39.25 ± 7.21**	**39.58 ± 4.62**	**76.31 ± 7.66**	**80.92 ± 5.61**	0.43476	< 0.00001	0.36415
**Alanine**	6.39 ± 0.98 ^a^	1.46 ± 0.12 ^b^	2.39 ± 0.22 ^a^	2.43 ± 0.17 ^a^	< 0.00001	-	-
**Asparagine**	3.03 ± 0.93 ^a^	1.51 ± 0.60 ^b^	4.71 ± 0.28 ^a^	4.93 ± 0.37 ^a^	0.00392	-	-
**Aspartic Acid**	1.80 ± 0.28 ^a^	2.18 ± 0.21 ^b^	3.44 ± 0.11 ^a^	3.65 ± 0.24 ^b^	0.33914	< 0.00001	0.04164
**Cysteine**	0.24 ± 0.02 ^a^	0.34 ± 0.07 ^b^	0.32 ± 0.02 ^a^	0.32 ± 0.02 ^a^	0.00981	-	-
**Glutamine**	20.61 ± 5.34 ^a^	6.26 ± 0.92 ^b^	33.23 ± 1.45 ^a^	35.55 ± 2.68 a	< 0.00001	-	-
**Glutamic acid**	4.56 ± 0.75 ^a^	6.27 ± 1.38 ^b^	5.3 ± 0.32 ^a^	6.30 ± 0.51 ^a^	0.08054	0.11181	0.03583
**Glycine**	0.85 ± 0.23 ^a^	0.96 ± 0.14 ^b^	1.44 ± 0.06 ^a^	1.59 ± 0.11 ^a^	0.64644	< 0.00001	0.04943
**Proline**	4.77 ± 0.82 ^a^	1.43 ± 0.13 ^b^	3.27 ± 0.36 ^a^	3.48 ± 0.29 ^a^	< 0.00001	-	-
**Serine**	1.33 ± 0.34 ^a^	3.08 ± 1.53 ^b^	2.23 ± 0.22 ^a^	2.34 ± 0.11 ^b^	0.03431	-	-
**Tyrosine**	0.59 ± 0.1 ^a^	0.88 ± 0.17 ^b^	1.33 ± 0.13 ^a^	1.44 ± 0.11 ^b^	0.15918	< 0.00001	0.00568
**ΣNEAA**	**44.17 ± 8.78 **^**a**^	**24.38 ± 2.27 **^**b**^	**58.44 ± 5.66 **^**a**^	**62.02 ± 4.65 **^**a**^	0.00013	**-**	**-**
**EAA/NEAA**	0.89 ± 0.09 ^a^	1.64 ± 0.18 ^b^	1.31 ± 0.01 ^a^	1.31 ± 0.01 ^a^	< 0.00001	**-**	**-**
**ΣFAA**	**95.12 ± 5.43 **^**a**^	**70.28 ± 4.95 **^**b**^	**141.52 ± 5.43 **^**a**^	**150.05 ± 4.95 **^**a**^	0.00483	**-**	**-**

The sum of NEAA was significantly influenced by the treatment × age interaction (p < 0.0001). When this model was broken down to evaluate the effect of treatment at each age, a significant difference was evident between treatments at the fertilized egg stage, where eggs from CPE batches had almost the double amount of NEAA compared to SPE batches, while no difference was observed in larvae at 8 dph. Similarly, among the NEAA, alanine, asparagine, cysteine, glutamine, proline, and serine were also significantly affected by the treatment × age interaction; thus, the model was decomposed to test the effect of treatment at each stage. A series of one-way ANOVAs were performed for these EAA, also revealing a significant difference between treatments at the fertilized egg stage (p < 0.001), but no difference at 8 dph. Aspartic acid, glycine, and tyrosine were influenced by both treatment (p < 0.0001) and age (p < 0.05). These NEAAs increased with age and were higher in SPE eggs and 8 dph larvae compared to CPE. Glutamic acid was the only FAA influenced exclusively by treatment (p = 0.035).

The ratio EAA/NEAA was influenced by the treatment × age interaction (p < 0.0001). Once more, the model was broken down to evaluate the effect of maternal treatment at each age, showing that the ratio was significantly different among treatment groups at the egg stage (p < 0.00001), but not for 8 dph larvae.

### Fatty acid analysis

The fatty acid composition in fertilized eggs (4 hpf) and developing yolk-sac larvae is presented in Table 3. The highest represented group was MUFAs, followed by SFAs and PUFAs. For all the fatty acids analysed in this study, no effect of treatment × age interaction was detected. Among the SFAs, the C14:0, C18:0, and C20:0 levels were affected by age (p < 0.01). Here, C14:0 showed an overall decreasing trend, while the proportion of the other two fatty acids increased throughout development. The proportions of the other SFAs analysed in this study (C15:0, C16:0, and C17:0) remained constant during the experimental period. The sum of MUFAs was significantly influenced by age, showing a decreasing trend throughout development. The sum of PUFAs was neither influenced by treatment nor age and remained constant between the fertilized egg stage and the end of the endogenous feeding stage. Levels of the essential fatty acids, EPA, ARA, and DHA, showed a significantly (p < 0.01) increasing trend during development, while the sum of *n-3* fatty acids significantly (p = 0.0012) increased between hatch (0 dph) and 13 dph. The sum of *n-6* was not affected by age or treatment but remained constant throughout the experimental period. The ratio EPA/ARA was significantly affected by age (p < 0.00001), where it decreased with development, while the opposite trend was apparent for the ratio DHA/EPA. The sum of fatty acids (ΣFA) remained constant throughout the experimental time.

**Table 3:** – Mean (± SD) proportion of main fatty acids, calculated as a percentage of total lipid in European eel fertilized eggs (4 hpf) and larvae (0, 8, and 13 dph)

*Fatty acids*	Egg	0 dph	8 dph	13 dph
*C14:0*	2.88 ± 0.54 ^c^	2.01 ± 0.78 ^b^	1.50 ± 0.23 ^b^	1.11 ± 0.19 ^a^
*C15:0*	0.33 ± 0.11	0.23 ± 0.14	0.25 ± 0.08	0.27 ± 0.04
*C16:0*	19.72 ± 1.69	22.54 ± 3.67	21.67 ± 3.18	25.26 ± 4.00
*C17:0*	0.57 ± 0.11	0.52 ± 0.11	0.51 ± 0.06	0.57 ± 0.07
*C18:0*	4.63 ± 0.40 ^a^	6.19 ± 2.00 ^b^	4.95 ± 0.48 ^a^	7.40 ± 0.63 ^c^
*C20:0*	0.21 ± 0.07 ^**ab**^	0.18 ± 0.13 ^a^	0.28 ± 0.09 ^**ab**^	0.50 ± 0.12 ^b^
***ΣSFA***	**28.03 ± 1.44** ^**ab**^	**31.43 ± 4.32** ^**ab**^	**29.15 ± 3.46** ^**a**^	**34.92 ± 4.33** ^**b**^
*C16:1*	8.93 ± 0.82 ^d^	7.93 ± 1.28 ^c^	6.41 ± 0.92 ^b^	4.79 ± 0.74 ^a^
*C17:1*	0.95 ± 0.17^c^	0.76 ± 0.23 ^b^	0.75 ± 0.09 ^b^	0.62 ± 0.07 ^a^
*C18:1*	34.21 ± 4.11^bc^	37.60 ± 3.63^bc^	32.33 ± 2.79 ^b^	28.38 ± 3.84 ^a^
*C20:1*	1.19 ± 0.23 ^b^	1.04 ± 0.29 ^b^	0.81 ± 0.18 ^a^	0.74 ± 0.15 ^a^
***ΣMUFA***	**45.28 ± 3.46 **^**c**^	**47.34 ± 3.57**^**c**^	**40.30 ± 6.03 **^**b**^	**34.53 ± 4.39** ^a^
*C18:2*	2.32 ± 0.53	2.62 ± 0.32	2.60 ± 0.25	2.39 ± 0.41
*C18:3*	0.11 ± 0.08	0.08 ± 0.05	0.08 ± 0.05	0.08 ± 0.05
*C20:2*	0.20 ± 0.10	0.14 ± 0.09	0.17 ± 0.06	0.17 ± 0.11
*C20:3*	0.22 ± 0.08	0.16 ± 0.11	0.23 ± 0.08	0.25 ± 0.12
***ΣPUFA***	**2.58 ± 1.09**	**2.34 ± 1.38**	**3.01 ± 0.34**	**2.82 ± 0.66**
*C20:4 – ARA*	1.93 ± 0.61 ^a^	2.11 ± 0.83 ^a^	3.86 ± 0.50 ^b^	6.58 ± 1.42 ^c^
*C20:5 – EPA*	1.46 ± 0.41 ^a^	1.47 ± 0.54 ^a^	1.88 ± 0.18 ^b^	2.33 ± 0.35 ^c^
*C22:6—DHA*	3.92 ± 1.06 ^a^	3.67 ± 1.47 ^a^	6.08 ± 1.50 ^b^	8.56 ± 2.63 ^c^
*Σn-3*	5.47 ± 1.34 ^a^	5.20 ± 1.95 ^a^	8.02 ± 1.46 ^b^	10.94 ± 2.98 ^c^
*Σn-6*	3.77 ± 0.81	4.09 ± 0.79	4.48 ± 0.30	4.72 ± 0.57
*n-3/n-6*	0.71 ± 0.15 ^a^	0.89 ± 0.37 ^a^	0.58 ± 0.13 ^b^	0.46 ± 0.13 ^c^
*EPA/ARA*	0.74 ± 0.58^d^	0.70 ± 0.76^c^	0.48 ± 0.84^b^	0.35 ± 0.76 ^a^
*DHA/EPA*	2.76 ± 0.09 ^ab^	2.52 ± 0.13 ^a^	3.26 ± 0.05 ^b^	3.61 ± 0.05 ^bc^
***ΣFA***	**87.75 ± 4.41**	**85.03 ± 5.28**	**84.62 ± 5.32**	**89.77 ± 5.26**

*SFA: saturated fatty acids, MUFA: mono-unsaturated fatty acids, PUFA: poly-unsaturated fatty acid, ARA: arachidonic acid, EPA: eicosapentaenoic acid, DHA: docosahexaenoic acid. Different letters represent significant differences (p* < *0.05)*

## Discussion

The present study assessed differences in quality and biochemical composition of European eel offspring produced via two alternative female treatment protocols (CPE *vs.* SPE). On the one side, CPE females produced eggs containing a higher amount of total lipids and free amino acids, while on the other, SPE females produced eggs showing a higher percentage of buoyant eggs and fertilization rate. The latter indicates that other maternally derived components than nutrients, such as mRNAs and steroid hormones, present in the egg cytoplasm, might govern the essential processes during early embryogenesis, as also recently suggested for European eel (Kottmann et al. 2020a; 2021).

Moreover, larvae from SPE females were larger at hatch than CPE. Considering the “bigger is better hypothesis” (Bailey and Houde 1989), having a larger body area at hatch is generally considered an advantage and has been related to higher survival for example in European sardine, *Sardina pilchardus* (Garrido et al. 2015), and capelin, *Mallotus villosus* (Chambers et al. 1989). However, this initial advantage did not translate into benefits neither in body area at 13 dph nor growth rate throughout the experimental period. In fact, the oil-droplet utilization (ODU) was higher in the originally smaller CPE larvae with smaller oil droplets, indicating an efficient usage of energy reserves, as CPE and SPE larvae grew to similar sizes at the end of the yolk-sac stage. In this regard, the oil-droplet (and/or yolk) utilization efficiency seems to not only depend on environmental conditions such as temperature, salinity, and oxygen (Kamler 2008), but also maternally derived hormones, such as testosterone and cortisol, as shown in species such as damselfish, *Pomacentrus amboinensis* (McCormick 1999)*.*

It is notable that most of the differences between treatments were observed at the egg stage and/or shortly after hatch, while they were no longer detectable during later larval development. At this point, larvae possessed similar total lipid and protein amounts, concentration of free amino acids as well as body and oil-droplet area. This progressive reduction of maternal hormonal treatment effects may be related to the shift from early ontogenetic regulation by maternal factors (present in the egg cytoplasm) towards the organisms own regulation pathways, a process called maternal-to-zygotic transition, commonly occurring after the mid-blastula stage (Lee et al. 2014). Kottmann et al. (2020b) compared offspring from farm-raised females with wild-caught females and found an increased mortality during maternal-to-zygotic transition of egg batches scoring low initial quality parameters. Thus, regardless of reproduction methods or female origin, only larvae endowed with adequate energy reserves and sufficient nutrients appeared to meet the stage-specific requirements, surviving, and developing successfully. Failure to meet these requirements causes morphological deformities (Gwak and Tanaka 2001; Kjørsvik et al. 1991) and leads to high mortality (Dou et al. 2002; Houde 1974).

The two experimental groups of larvae exhibited common paths of utilization of the lipid, protein, fatty acid, and free amino acid resources. Despite the high variability in offspring quality, the gradual decrease in the amounts of total lipid and MUFA available at different yolk-sac stages was similar and in line with observations for Japanese eel, *Anguilla japonica* (Furuita et al. 2006). This indicates that lipids and MUFAs were the main sources of energy during embryogenesis and early development of larvae. Beyond 8 dph, the lipid and MUFA content remained constant, suggesting that larvae at this stage are genetically pre-programmed to explore and exploit exogenous sources of lipids and fatty acids to meet their energy requirements. In fact, eel larvae start the expression of genes related to the main digestive enzymes (amylase, lipase, and trypsin) between 4 and 8 dph, marking the onset of molecular ontogeny of the feeding mechanism and the inclination to receive and process exogenous feed (Politis et al. 2018b). Among MUFAs, the oleic acid (C18:1) was the fatty acid with the highest concentration. This fatty acid, together with palmitic acid, DHA and EPA are considered among the essential fatty acids (Izquierdo 1996). Moreover, oleic acid and the other MUFAs have been regarded as main sources of energy during early development of marine fish larvae (van der Meeren et al. 2008). While MUFAs are consumed during the yolk-sac stage, palmitic acid and the other SFAs were not used, but preserved at later developmental stages. In addition, the amount of PUFA remained unchanged with larval development, as observed also in other marine fish species, where this class of fatty acids is preferentially incorporated into structural lipids in larval tissue (Wiegand 1996). This included that DHA, EPA, and ARA, playing a vital role in the physiology of fish larvae, were retained during the yolk-sac period. This pattern has been described also for other marine larvae such as Atlantic halibut (Rønnestad et al. 1995), turbot, *Scophthalmus maximus* (Rainuzzo et al. 1994), Atlantic cod (van der Meeren et al. 1993), and gilthead seabream, *Sparus aurata* (Rodriguez et al. 1994). Since eel larvae (as most marine fish species) are incapable of de novo synthesis of these fatty acids, EFAs are usually the last class of fatty acids used as energy source (Bell and Sargent 2003).

The eggs of European eel contained approximately 35% of protein, which is similar to previous reports on European eel and other eel species such as Japanese, American, *Anguilla rostrata,* and short-finned, *Anguilla australis* eel (Heinsbroek et al. 2013). In American shad, *Alosa sapidissima* (Liu et al. 2018), brill, *Scophthalmus rhombus L*. (Cruzado et al. 2013), and cobia, *Rachycentron canadum* (Huang et al. 2021) as well as Japanese eel (Ohkubo et al. 2008), the concentration of protein after hatching has been reported to decrease due to high demand for energy, besides being used as building blocks during embryonic development. In the present study, the protein content of the fertilized eggs was similar to the content of 13-day-old larvae and thus was retained during the entire endogenous feeding period. We can speculate that European eel embryos and larvae utilize the yolk protein to grow and develop their body, transforming from being part of the yolk reserve to be part of the body, without being used for energetic purposes.

Moreover, FAAs slightly increased from 7–10% at the egg stage to 14–15% in larvae at 8 dph, levels which are similar to Japanese eel, but tenfold lower than Atlantic cod and threefold lower than turbot (Fyhn 1990). As for total FAAs, the free amino acid pool was maintained between the fertilized egg stage and the middle-end of the yolk-sac larval stage. This differed from previous studies on Japanese and European eel (Heinsbroek et al. 2013; Ohkubo et al 2008), where FAAs decreased most between hatch and 4 dph. On the other hand, the FAA profile of the eggs in the present study was similar to that of Japanese eel eggs, where glutamine (Gln) shows highest concentration, followed by arginine and alanine (Ohkubo et al. 2008). Glutamine is considered a NEAA, but it has proven to be a fundamental requirement for the synthesis of glycosaminoglycan (GAG) (Watford 2015), which is the basic material of the gelatinous matrix congrid eel, *Ariosoma balearicum* larvae are made of (Donnelly et al. 1995). As discussed by Ohkubo et al. (2008), non-essential amino acids could be synthetized or converted within the egg/larvae due to specific needs and the author reckoned that Gln could be used for protein synthesis or as an energy source. Considering that the amount of Gln increased at 8 dph, it is possible that Gln has been synthesized to assemble the gelatinous matrix, typical for the leptocephalus larvae of the anguilliform species. However, further investigations are necessary to clarify the amino acid modifications during yolk proteolysis in European eel.

In conclusion, female broodstock treated with either of the two hormonal treatments (SPE and CPE) were able to produce viable offspring. The SPE protocol provided the highest quality of eggs in terms of higher fertilization rate and size (body area) at hatch. However, CPE females produced offspring containing higher amounts of lipids and FAAs, which could potentially be of benefit to larval fitness in case of adverse environmental conditions or prolonged starvation. Nevertheless, after hatching, the differences between treatments levelled-out and were no longer detectable among larvae surviving to the first-feeding stage. Furthermore, eel larvae utilized MUFAs and lipids as main sources of energy until day 8, when they need to start switching to exogenous feeding to meet energy demands and constituent requirements. On the contrary, EFAs were not spent, which likely is due to their importance in regulating immune and inflammatory responses as well as cellular metabolism. Similarly, also FAAs were retained, probably to be used throughout later development.

### Statements and Declarations

## Conflict of interest:

The authors declare no competing interests.

## Ethics approval

All fish were handled in accordance with the European Union regulations concerning the protection of experimental animals (Directive 2010/63/EU). Eel experimental protocols were approved by the Animal Experiments Inspectorate (AEI), Danish Ministry of Food, Agriculture and Fisheries (permit number: 2015–15-0201–00,696). Submergence in an aqueous solution of ethyl p-aminobenzoate (benzocaine, 20 mg L^−1^, Sigma-Aldrich, Germany) was applied to anaesthetize individual eels in relation to tagging (all fishes), ovarian biopsy, and stripping of gametes (females) and to euthanize individuals after stripping (females) or at the end of the experiment (males). For larvae, tricaine methanesulfonate (MS-222, 25 mg L^−1^, Sigma-Aldrich, Germany) was used to anaesthetize larvae before photography and to euthanize sampled larvae before preservation.

**Consent to participate:** All authors consent to participate.

**Consent for publication:** All authors approved the submitted version of this manuscript.

## Availability of data and material:

The data that support the findings of this study are available from the corresponding author upon reasonable request.

## Code availability:

Not applicable.

## References

[CR1] AOAC (2006) Official Methods of Analysis. 18^th^ Edition, Association of Official Analytical Chemists, Gaithersburgs, MD.

[CR2] Bailey KM, Houde ED (1989). Predation on eggs and larvae of marine fishes and the recruitment problem. Adv Mar Biol.

[CR3] Bell JG, Sargent JR (2003). Arachidonic acid in aquaculture feeds: current status and future opportunities. Aquaculture.

[CR4] Benini E, Politis SN, Kottmann JS, Butts IA, Sørensen SR, Tomkiewicz J (2018). Effect of parental origin on early life history traits of European eel. Reprod Domest Anim.

[CR5] Bligh EG, Dyer WJ (1959). A rapid method of total lipid extraction and purification. Can J Biochem Physiol.

[CR6] Bobe J (2015). Egg quality in fish: Present and future challenges. Anim Front.

[CR7] Bobe J, Labbé C (2010). Egg and sperm quality in fish. Gen Comp Endocrinol.

[CR8] Brooks S, Tyler CR, Sumpter JP (1997). Egg quality in fish: What makes a good egg?. Reviews in Fish Biology and Fishery.

[CR9] Butts IAE, Sørensen SR, Politis SN, Pitcher TE, Tomkiewicz J (2014). Standardization of fertilization protocols for the European eel, *Anguilla anguilla*. Aquaculture.

[CR10] Chambers RC, Leggett WC, Brown JA (1989). Egg size, female effects, and the correlations between early life history traits of capelin, *Mallotus villosus*: an appraisal at the individual level. Fish Bull.

[CR11] Conceição L, Tandler A (2018) Success factors for fish larval production. John Wiley & Sons

[CR12] Cruzado IH, Rodríguez E, Herrera M, Lorenzo A, Almansa E (2013). Changes in lipid classes, fatty acids, protein and amino acids during egg development and yolk‐sac larvae stage in brill (*Scophthalmus rhombus L*.). Aquaculture Research.

[CR13] da Silva FFG, Jacobsen C, Kjørsvik E, G. Støttrup J, Tomkiewicz J.  (2018). Oocyte and egg quality indicators in European eel: Lipid droplet coalescence and fatty acid composition. Aquaculture.

[CR14] Donnelly J, Torres JJ, Crabtree RE (1995). Proximate composition and nucleic acid content of premetamorphic leptocephalus larvae of the congrid eel *Ariosoma balearicum*. Mar Biol.

[CR15] Dou S, Masuda R, Tanaka M, Tsukamoto K (2002). Feeding resumption, morphological changes and mortality during starvation in Japanese flounder larvae. J Fish Biol.

[CR16] FAO (2020). The State of World Fisheries and Aquaculture 2020.

[CR17] Finn RN, Henderson JR, Fyhn HJ (1994). Physiological energetics of developing embryos and yolk-sac larvae of Atlantic cod (*Gadus morhua*). II. Lipid metabolism and enthalpy balance. Marine Biology.

[CR18] Folch J, Lees M, Stanley GS (1957). A simple method for the isolation and purification of total lipids from animal tissues. J Biol Chem.

[CR19] Furuita H, Unuma T, Nomura K, Tanaka H, Okuzawa K, Sugita T, Yamamoto T (2006). Lipid and fatty acid composition of eggs producing larvae with high survival rate in the Japanese eel. J Fish Biol.

[CR20] Fyhn HJ (1990). First feeding of marine fish larvae-are free amino acids the source of energy. Aquaculture.

[CR21] Garrido S, Ben-Hamadou R, Santos AMP, Ferreira S, Teodósio MA, Cotano U, Irigoien X, Peck MA, Saiz E, Re P (2015). Born small, die young: Intrinsic, size-selective mortality in marine larval fish. Sci Rep.

[CR22] Gwak WS, Tanaka M (2001). Developmental change in RNA: DNA ratios of fed and starved laboratory-reared Japanese flounder larvae and juveniles, and its application to assessment of nutritional condition for wild fish. J Fish Biol.

[CR23] Heinsbroek LTN, Støttrup JG, Jacobsen C, Corraze G, Kraiem MM, Holst LK, Tomkiewicz J, Kaushik SJ (2013). A review on broodstock nutrition of marine pelagic spawners: the curious case of the freshwater eels (Anguilla spp.). Aquac Nutr.

[CR24] Houde ED (1974). Effects of temperature and delayed feeding on growth and survival of larvae of three species of subtropical marine fishes. Mar Biol.

[CR25] Huang JS, Amenyogbe E, Chen G, Wang WZ (2021). Biochemical composition and activities of digestive and antioxidant enzymes during the egg and yolk-sac larval development of the cobia (*Rachycentron canadum*). Aquac Res.

[CR26] Izquierdo MS (1996). Essential fatty acid requirements of cultured marine fish larvae. Aquac Nutr.

[CR27] Izquierdo, M. (2005) Essential fatty acid requirements in Mediterranean fish species. Cahiers Options Méditerranéennes.

[CR28] Kamler E (2008). Resource allocation in yolk-feeding fish. Rev Fish Biol Fisheries.

[CR29] Keppel, G. (1991) Design and analysis: A researcher’s handbook. Prentice-Hall, Inc.

[CR30] Kjørsvik E, Van der Meeren T, Kryvi H, Arnfinnson J, Kvenseth PG (1991). Early development of the digestive tract of cod larvae, Gadus morhua L., during start‐feeding and starvation. Journal of fish Biology.

[CR31] Kottmann JS, Jørgensen MG, Bertolini F, Loh A, Tomkiewicz J (2020). Differential impacts of carp and salmon pituitary extracts on induced oogenesis, egg quality, molecular ontogeny and embryonic developmental competence in European eel. PLoS ONE.

[CR32] Kottmann, J.S., Tomkiewicz, J., Butts, I.A., Lund, I., Jacobsen, C., Støttrup, J.G., Holst, L. (2020b) Effects of essential fatty acids and feeding regimes on egg and offspring quality of European eel: Comparing reproductive success of farm-raised and wild-caught broodstock. Aquaculture, 529, 735581.

[CR33] Kottmann, J.S., Tveiten, H., Miest, J.J.,Tomkiewicz, J. (2021) Sex steroid dynamics and mRNA transcript profiles of growth-and development-related genes during embryogenesis following induced follicular maturation in European eel. General and Comparative Endocrinology,113854. 10.1016/j.ygcen.2021.113854 34265345

[CR34] Koumpiadis, P., Sganga, D. E., Politis, S. N., Gallego, V., Butts, I. A., Asturiano, J. F., Tomkiewicz J. (2021). Sperm production and quality in European eel (*Anguilla anguilla*) in relation to hormonal treatment. Reproduction in Domestic Animals. 10.1111/rda.14011 34478180

[CR35] Lee MT, Bonneau AR, Giraldez AJ (2014). Zygotic genome activation during the maternal-to-zygotic transition. Annu Rev Cell Dev Biol.

[CR36] Li, H., and Zhang, S. (2017) Functions of vitellogenin in eggs. Oocytes, 389–401. 10.1007/978-3-319-60855-6_17 28779327

[CR37] Liu Z, Gao X, Yu J, Wang Y, Guo Z, Huang B, Liu B, Hong L (2018). Changes of protein and lipid contents, amino acid and fatty acid compositions in eggs and yolk-sac larvae of American shad (*Alosa sapidissima*). Journal of Ocean University of China.

[CR38] Lubzens E, Young G, Bobe J, Cerdà J (2010). Oogenesis in teleosts: how fish eggs are formed. Gen Comp Endocrinol.

[CR39] McCormick MI (1999). Experimental test of the effect of maternal hormones on larval quality of a coral reef fish. Oecologia.

[CR40] Migaud H, Bell G, Cabrita E, McAndrew B, Davie A, Bobe J, Herraez MP, Carrillo M (2013). Gamete quality and broodstock management in temperate fish. Rev Aquac.

[CR41] Mordenti, O., Casalini, A., Parmeggiani, A., Emmanuele, P., Zaccaroni, A. (2019) December. Captive breeding of the European eel: Italian. In Eels Biology, Monitoring, Management, Culture and Exploitation: Proceedings of the First International Eel Science Symposium. 5m Books Ltd.

[CR42] Mylonas, C., and Zohar, Y. (2010) Controlling fish reproduction in aquaculture. In New Technologies in Aquaculture, 109–142. Woodhead Publishing.

[CR43] Nagahama Y, Yamashita M (2008). Regulation of oocyte maturation in fish. Dev Growth Differ.

[CR44] Ohkubo N, Sawaguchi S, Nomura K, Tanaka H, Matsubara T (2008). Utilization of free amino acids, yolk protein and lipids in developing eggs and yolk-sac larvae of Japanese eel *Anguilla japonica*. Aquaculture.

[CR45] Palstra AP, Cohen EGH, Niemantsverdriet PRW, Van Ginneken VJT, Van den Thillart GEEJM (2005). Artificial maturation and reproduction of European silver eel: development of oocytes during final maturation. Aquaculture.

[CR46] Peñaranda DS, Marco-Jiménez F, Pérez L, Gallego V, Mazzeo I, Vicente JS, Jover M, Asturiano JF (2010). Evaluation of different diluents for short-term storage of European eel sperm under air-limited conditions. J Appl Ichthyol.

[CR47] Pérez L, Aturiano JF, Tomás A, Zegrari S, Barrera R, Espinós FJ, Navarro JC, Jover M (2000). Induction of maturation and spermiation in the male European eel: assessment of sperm quality throughout treatment. J Fish Biol.

[CR48] Politis SN, Butts IA, Tomkiewicz J (2014). Light impacts embryonic and early larval development of the European eel, *Anguilla anguilla*. J Exp Mar Biol Ecol.

[CR49] Politis SN, Mazurais D, Servili A, Zambonino-Infante JL, Miest JJ, Sørensen SR, Tomkiewicz J, Butts IA (2017). Temperature effects on gene expression and morphological development of European eel. Anguilla Anguilla Larvae PLOS One.

[CR50] Politis SN, Mazurais D, Servili A, Zambonino-Infante JL, Miest JJ, Tomkiewicz J, Butts IA (2018). Salinity reduction benefits European eel larvae: Insights at the morphological and molecular level. PLoS ONE.

[CR51] Politis SN, Sørensen SR, Mazurais D, Servili A, Zambonino-Infante JL, Miest JJ, Clemmesen CM, Tomkiewicz J, Butts IA (2018). Molecular ontogeny of first-feeding European eel larvae. Front Physiol.

[CR52] Politis SN, Syropoulou E, Benini E, Bertolini F, Sørensen SR, Miest JJ, Butts IAE, Tomkiewicz J (2021). Performance thresholds of hatchery produced European eel larvae reared at different salinity regimes. Aquaculture.

[CR53] Rainuzzo JR, Reitan KI, Jørgensen L, Olsen Y (1994). Lipid composition in turbot larvae fed live feed cultured by emulsions of different lipid classes. Comp Biochem Physiol A Physiol.

[CR54] Reading BJ, Andersen LK, Ryu YW, Mushirobira Y, Todo T, Hiramatsu N (2018). Oogenesis and egg quality in finfish: yolk formation and other factors influencing female fertility. Fishes.

[CR55] Rodriguez C, Perez JA, Izquierdo MS, Mora J, Lorenzo A, Fernandez‐Palacios H (1994). Essential fatty acid requirements of larval gilthead sea bream, Sparus aurata (L.). Aquaculture Research.

[CR56] Rønnestad I, Groot EP, Fyhn HJ (1993). Compartmental distribution of free amino acids and protein in developing yolk-sac larvae of Atlantic halibut (*Hippoglossus hippoglossus*). Mar Biol.

[CR57] Rønnestad I, Finn RN, Lie Ø, Lein I (1995). Compartmental changes in the contents of total lipid, lipid classes and their associated fatty acids in developing yolk‐sac larvae of Atlantic halibut, Hippoglossus hippoglossus (L.). Aquaculture Nutrition.

[CR58] Rønnestad I, Thorsen A, Finn RN (1999). Fish larval nutrition: a review of recent advances in the roles of amino acids. Aquaculture.

[CR59] Rønnestad I, Yúfera M, Ueberschär B, Ribeiro L, Sæle Ø, Boglione C (2013). Feeding behaviour and digestive physiology in larval fish: current knowledge, and gaps and bottlenecks in research. Rev Aquac.

[CR60] Sargent J, McEvoy L, Estevez A, Bell G, Bell M, Henderson J, Tocher D (1999). Lipid nutrition of marine fish during early development: current status and future directions. Aquaculture.

[CR61] Schmitz M, Aroua S, Vidal B, Le Belle N, Elie P, Dufour S (2005). Differential regulation of luteinizing hormone and follicle-stimulating hormone expression during ovarian development and under sexual steroid feedback in the European eel. Neuroendocrinology.

[CR62] Seoka M, Yamada S, Kumai H (2004). Free amino acids in Japanese eel eggs obtained by hormonal inducement. J Fish Biol.

[CR63] Sørensen SR, Gallego V, Pérez L, Butts IAE, Tomkiewicz J, Asturiano JF (2013). Evaluation of methods to determine sperm density for the European eel. Anguilla Anguilla Reproduction in Domestic Animals.

[CR64] Sørensen SR, Skov PV, Lauesen P, Tomkiewicz J, Bossier P, De Schryver P (2014). Microbial interference and potential control in culture of European eel (*Anguilla anguilla*) embryos and larvae. Aquaculture.

[CR65] Sørensen SR, Butts IAE, Munk P, Tomkiewicz J (2016). Effects of salinity and sea salt type on egg activation, fertilization, buoyancy and early embryology of European eel, *Anguilla anguilla*. Zygote.

[CR66] Sørensen SR, Tomkiewicz J, Munk P, Butts IA, Nielsen A, Lauesen P, Graver C (2016). Ontogeny and growth of early life stages of captive-bred European eel. Aquaculture.

[CR67] Støttrup JG, Jacobsen C, Tomkiewicz J, Jarlbæk H (2013). Modification of essential fatty acid composition in broodstock of cultured E uropean eel *Anguilla anguilla* L. Aquac Nutr.

[CR68] Støttrup JG, Tomkiewicz J, Jacobsen C, Butts IAE, Holst LK, Krüger-Johnsen M, Graver C, Lauesen P, Fontagné-Dicharry S, Heinsbroek LTN, Corraze G (2016). Development of a broodstock diet to improve developmental competence of embryos in European eel. Anguilla Anguilla Aquaculture Nutrition.

[CR69] Suzuki K, Nagahama Y, Kawauchi H (1998). Steroidogenic activities of 2 distinct salmon gonadotropins. Gen Comp Endocrinol.

[CR70] Tocher DR (2010). Fatty acid requirements in ontogeny of marine and freshwater fish. Aquac Res.

[CR71] Tomkiewicz, J., Politis, S.N., Sørensen, S.R., Butts, I.A., Kottmann, J.S. (2019) European eel – an integrated approach to establish eel hatchery technology in Denmark. In A. Don, & P. Coulson (Eds.), Eels - Biology, Monitoring, Management, Culture and Exploitation: Proceedings of the First International Eel Science Symposium (pp. 340–374). 5M Publishing.

[CR72] Tomkiewicz, J. (2012) Reproduction of European Eel in Aquaculture (REEL): consolidation and new production methods.

[CR73] Tyler CR, Sumpter JP (1996). Oocyte growth and development in teleosts. Rev Fish Biol Fisheries.

[CR74] van der Meeren T, Wilhelmsen S, Klungsosyr J, Kvenseth PG (1993). Fatty acid composition of unfed cod larvae *Gadus morhua* L. and cod larvae feeding on natural plankton in large enclosures. Journal of the World Aquaculture Society.

[CR75] van der Meeren T, Olsen RE, Hamre K, Fyhn HJ (2008). Biochemical composition of copepods for evaluation of feed quality in production of juvenile marine fish. Aquaculture.

[CR76] Vidal B, Pasqualini C, Le Belle N, Holland MCH, Sbaihi M, Vernier P, Zohar Y, Dufour S (2004) Dopamine inhibits luteinizing hormone synthesis and release in the juvenile European eel: a neuroendocrine lock for the onset of puberty. Biol Reprod 71(5):1491–1500 10.1095/biolreprod.104.030627 15229141

[CR77] Watford M (2015). Glutamine and glutamate: Nonessential or essential amino acids?. Animal Nutrition.

[CR78] Weber, G. M., and Lee, C. S. (2014) Current and future assisted reproductive technologies for fish species. Current and Future Reproductive Technologies and World Food Production, 33–76. 10.1007/978-1-4614-8887-3_3 24170354

[CR79] Wiegand MD (1996). Composition, accumulation and utilization of yolk lipids in teleost fish. Rev Fish Biol Fisheries.

